# Plant Phylogeny and Growth Form as Drivers of the Altitudinal Variation in Woody Leaf Vein Traits

**DOI:** 10.3389/fpls.2019.01735

**Published:** 2020-02-05

**Authors:** Ruili Wang, Haoxuan Chen, Xinrui Liu, Zhibo Wang, Jingwen Wen, Shuoxin Zhang

**Affiliations:** ^1^ College of Forestry, Northwest A&F University, Yangling, China; ^2^ Qinling National Forest Ecosystem Research Station, Huoditang, China; ^3^ State Key Laboratory of Soil Erosion and Dryland Farming on the Loess Plateau, Northwest A&F University, Yangling, China; ^4^ Institute of Soil and Water Conservation, Chinese Academy of Sciences and Ministry of Water Resources, Yangling, China

**Keywords:** leaf venation, phylogeny, plant functional type, climate, elevational gradient

## Abstract

Variation in leaf veins along environmental gradients reflects an important adaptive strategy of plants to the external habitats, because of their crucial roles in maintaining leaf water status and photosynthetic capacity. However, most studies concentrate on a few species and their vein variation across horizontal spatial scale, we know little about how vein traits shift along the vertical scale, e.g., elevational gradient along a mountain, and how such patterns are shaped by plant types and environmental factors. Here, we aimed to investigate the variation in leaf vein traits (i.e., vein density, VD; vein thickness, VT; and vein volume per unit leaf area, VV) of 93 woody species distributed along an elevational gradient (1,374–3,375 m) in a temperate mountain in China. Our results showed that altitude-related trends differed between growth forms. Tree plants from higher altitudes had lower VD but higher VT and VV than those from lower altitude; however, the opposite tend was observed in VD of shrubs, and no significant altitudinal changes in their VT or VV. Plant phylogenetic information at the clade level rather than climate explained most of variation in three leaf vein traits (17.1–86.6% vs. <0.011–6.3% explained variance), supporting the phylogenetic conservatism hypothesis for leaf vein traits. Moreover, the phylogenetic effects on vein traits differed between trees and shrubs, with the vein traits of trees being relatively more conserved. Together, our study provides new picture of leaf vein variation along the altitude, and highlights the importance of taking plant phylogeny into consideration when discussing trait variation from an ecological to a biogeographic scale.

## Introduction

Leaf veins are composed of xylem and phloem cells that are embedded in parenchyma, or sometimes sclerenchyma, and are surrounded by bundle sheath cells (Sack and Scoffoni, 2013). The xylem of leaves transports nutrients and water, in order to facilitate leaf photosynthesis and transpiration, whereas the phloem exports leaf photosynthates and signal molecules to the rest of the plant (Sack et al., 2012; Sack and Scoffoni, 2013). Thus, the design and function of leaf venation, especially in regards to minor leaf veins (i.e., the high order veins in leaves), play key roles in plant growth, species assemblages in communities, and the energy, water and nutrient cycles of ecosystems (Boyce et al., 2009; Brodribb and Feild, 2010; Sack and Scoffoni, 2013; Griffin-Nolan et al., 2018). Among leaf vein traits, the total length of minor leaf vein per unit leaf area (e.g., vein density, VD) and vein thickness (VT) have attracted increasing interest in a wide range of fields (e.g., in agriculture, paleoecology and paleoclimatology, even in urban ecology) (Sack et al., 2012; Feild and Brodribb, 2013; Sack and Scoffoni, 2013), because of their importance in maintaining leaf water status and photosynthetic capacity (Brodribb et al., 2007; Beerling and Franks, 2010; Brodribb and Feild, 2010; Sack and Scoffoni, 2013).

Variation in leaf venation pattern is influenced at both the ontogenetic and evolutionary levels by a variety of climatic and environmental parameters (Uhl and Mosbrugger, 1999; Sack and Scoffoni, 2013; Blonder and Enquist, 2014). Plants can modify the geometry and structure of leaf venation to adapt to different hydraulic environments (Brodribb et al., 2010; de Boer et al., 2012; Blonder and Enquist, 2014). In drier regions, for example, the lower air humidity increases the diﬀerence between the water content of the leaf interior and the atmosphere, and thus the vapor pressure deficit increases, which eventually results in increased transpiration (Uhl and Mosbrugger, 1999; Schneider et al., 2018). In order to maintain a suitable leaf water supply-demand balance, plants can invest in higher VD to satisfy high transpirational demand (Brodribb et al., 2010; Sack and Scoffoni, 2013; Blonder and Enquist, 2014; Blonder et al., 2017). Meanwhile, VT decreases with increasing aridity, in order to minimize the cost of vein construction (Beerling and Franks, 2010) and to maintain vein spacing at levels that do not impede photosynthetic rate (Feild and Brodribb, 2013; Schneider et al., 2018). Thus, environmental factors that increase the transpiration of plants or decrease water availability tend to mediate greater VD and lower VT (Uhl and Mosbrugger, 1999; Sack and Scoffoni, 2013). Indeed, some data from short-term control experiments and observations in the field have confirmed the association between climate and VD by demonstrating that sun-adapted species and species from relatively dry or high-temperature climates tend to possess greater VD (Brodribb et al., 2010; Sack and Scoffoni, 2013; Blonder et al., 2017). However, based on the analysis of 238 herbarium specimens, Schneider et al. (2018) reported the weak overall relationships between vein traits and climatic variables. Additionally, based on a survey of 485 global species, Sack et al. (2012) emphasized that both larger leaves from moist regions and small leaves from dry regions may possess high VD. These studies suggest that trait–environment relationships can be obscured by different underlying processes, such as adaptive strategies and phylogenetic constraints, and, therefore, require further study (Blonder et al., 2017; Schneider et al., 2018).

Although some plants have demonstrated a capacity to modify the conductivity and density of leaf veins under different hydraulic condition, it seems that, in many clades of land plants, the topology of vein branching is relatively conservative and slow to evolve (Boyce et al., 2009; Brodribb et al., 2010; Sack and Scoffoni, 2013). Minor leaves veins, for example, have been reported to become denser and thinner during the radiation of angiosperms, whereas more basal angiosperm and non-angiosperm lineages (e.g., ferns and gymnosperms) generally possess relatively low VD values (Boyce et al., 2009; Feild et al., 2009; Feild and Brodribb, 2013). Moreover, significant phylogenetic signals have been detected among the VD, inter-vein maximum distance, and vascular bundle radii of both closely and distantly related phylogenetic clades (e.g., Zhang et al., 2012; Liu et al., 2015), indicating that variation in vein traits is constrained by phylogenetic background. If these minor leaf vein traits are phylogenetically conserved, leaf venation network would be less affected by the environmental factors and keep relatively stable under changing environmental conditions. By contrast, leaf venation and the relevant plant physiological process would vary with the changed environments. Therefore, to understand how vein trait variation is shaped by plant phylogeny and environmental factors is urgently needed to advance the understanding of plant adaptive strategies to fluctuating environments (McGill et al., 2006; Blonder and Enquist, 2014; Blonder et al., 2017). As an important criterion of plant classification, leaf vein traits should be relatively invariant for a given species, and thus we propose the leaf vein trait phylogenetic conservatism hypothesis, which means that differences in leaf vein networks can be mainly attributed to intrinsic evolutionary adaptations among major phylogenetic clades, with plants evolving denser and thinner minor leaf veins to counter the reduction in atmospheric CO_2_ concentrations that occurred during the Cretaceous (Brodribb and Feild, 2010; Feild and Brodribb, 2013; Sack and Scoffoni, 2013).

In addition, considerable differences in leaf vein traits are found in different growth forms. For example, mean VD is generally lowest in herbs and greatest in trees, with intermediate values in shrubs (Sack and Scoffoni, 2013). Moreover, their adaptation to severe habitats (e.g., drought) are different, with a general trend to decrease from drought-deciduous shrubs and ephemeral life-forms to trees with evergreen leaves (Woodward, 1993; Niinemets, 2001). Thus, we expect the phylogenetic conservation of vein traits may differ between trees and shrubs, with less phylogenetic conservatism in the vein traits of shrubs, owing to their greater capacity for environmental adaptation.

To test these hypothese, we examined leaf vein traits of 93 tree and shrub species along a 2,000-m altitudinal gradient in central China. These selected species encompassed broad phylogenetic lineages, which allowed the trait variation and its potential drivers to be investigated from a phylogenetic perspective. Three leaf vein traits that contribute to the hydraulic function and construction cost of leaves, namely VD, VT, and total vein volume per leaf area (VV) (Sack et al., 2012; Sack and Scoffoni, 2013), were selected for analysis.

## Materials and Methods

### Study Site and Sampling Methods

The present study was conducted on the northern slope of Taibai Mountain Nature Reserve (33° 49′N–34° 10′N, 107° 19′ E –107° 58′E) in Shaanxi Province, central China. The elevations of the nature reserve vary from 1,060 to 3,767.2 m, and Taibai Mountain is the highest mountain east of Qinghai–Tibet Plateau in mainland China. Along this altitude, the huge topographic and climatic differences result in a vertical zonation of major forest types. For example, in northern slope of Taibai Mountain, deciduous broadleaved forest dominated by *Quercus* spp. was the typical vegetation type below 2,300 m, whereas temperate birch forest, which was dominated by *Betula albosinensis* and *B. utilis*, was prominent from 2,300 to 2,800 m. Fir (*Abies fargesii*) forest and Larch (*Larix potaninii* var. *chinensis*) forest occurred at higher altitudes (2,600–3,000 m and 3,000–3,350 m, respectively) and formed a climate-induced treeline. Alpine shrubland, which was dominated by *Rhododendron capitatum*, was found above 3,350 m (**Supplementary Figure S1**) (Ren et al., 2006; Tang and Fang, 2006).

To investigate the leaf vein traits in typical vegetation zones, five sampling sites with four experimental plots (20 × 20 m plot for forest sites, 5 × 5 m plot for shrubland) at each vertical vegetation belt were established along the altitudinal gradients. In our sampling sites, the range of elevation is 1,374–3,375 m (a 2,000 m altitude gradient), which covers all typical vegetation types in Taibai Mountain (**Supplementary Table S1** and **Figure S1**). In each plot, 20 fully expanded sun leaves were collected from at least four individuals of each plant species, 93 woody species from 62 genera and 39 families were sampled in total. Some of the species occurred frequently, whereas others occurred at only one or two sites (**Supplementary Table S2**). Notably, except *L. potaninii* var. *chinensis*, all sampled conifers are evergreen trees. All of three evergreen angiosperm species in our dataset belong to Ericaceae family and shrub.

### Leaf Vein Observation

For each species, five leaves were randomly chosen for anatomical study. A 1-cm^2^ section was cut from each leaf without any primary veins. The leaf veins were then subject to chemical clearing (Sack et al., 2012; Blonder and Enquist, 2014). Briefly, the leaf samples were immersed in a 7% (w/v) solution of NaOH (in H_2_O); heated to 100°C for approximately 3 min, until the samples became transparent; rinsed in H_2_O for 30 min; transferred to 5% (w/v) NaOCl (in H_2_O) for 5 min; and rinsed again in H_2_O for 3 min. Next, the leaf samples were stained in 1% (w/v) safranin O for 15 min, in order to color the lignin-rich veins, and finally, the samples were mounted on microscope slides and photographed at 200× magnification using a Motic BA210 microscope (Motic Medical Diagnostic Systems, Co., Ltd., Xiamen, China). A total of 1260 leaf vein images were subject to analysis. Vein length and thickness (VT, μm) were measuring using digital images and Motic Images Plus 3.0 (Motic Medical Diagnostic Systems). Vein density (VD, mm mm^–2^) was measured as vein length per unit leaf area, and leaf vein volume (VV, mm^3^ mm^–2^) was calculated according to Sack et al. (2012):

$$\text{VV}=\pi \times {\left(\frac{\text{VT}}{2}\right)}^{2}\times VD$$

### Climatic Variables

Climatic variables for each sampling site, including mean annual temperature (MAT) and mean annual precipitation (MAP) were collected from the WorldClim global database with a spatial resolution of about 1 km^2^ (http://www.worldclim.org). Values of aridity index (AI) are downloaded from Global Aridity Index and Potential Evapotranspiration (ET0) Climate Database v2 (https://cgiarcsi.community/2019/01/24/global-aridity-index-and-potential-evapotranspiration-climate-database-v2/). Lower values of AI indicate a drier climate.

### Species Phylogeny

Species names were checked and standardized according to The Plant List (http://www.theplantlist.org/). Angiosperm order and family assignments were based on the Angiosperm Phylogeny Group IV classification (APG, 2016). A phylogenetic tree was constructed using the comprehensive species-level angiosperm phylogeny (Zanne et al., 2014) in phylomatic version 3 (http://phylodiversity.net/phylomatic/).

### Data Analysis

All analyses were conducted using the R 3.1.1 statistical platform (R Core Development Team, http://www.r-project.org/). Vein trait data were log_10_-transformed to obtain approximate normality and residual homogeneity.

Data of each species was first classified into different groups: growth form (GF, shrub and tree), leaf type (needled-leaves vs. evergreen and deciduous broadleaved species). To test for differences in vein traits of the various groups, both standard and phylogenetic one-way analyses of variance (ANOVA) were performed on the raw data, using the R function ‘aov’ with Tukey's significant difference test as well as the ‘phy.anova’ function in the R package ‘Geiger’. The bivariate analyses of VT–VD relationship was then performed using ordinary least squares (OLS) linear regressions. Differences in the slopes and elevations of these relationships between trees and shrubs were evaluated by standardized major axis estimation (SMA) estimation with the R package ‘smatr’. Phylogenetic generalized least squares (PGLS) analysis was also performed to evaluate the impacts of phylogenetic autocorrelation on the trait relationships.

To assess the phylogenetic conservatism of each trait, we tested for phylogenetic signal in all traits by performing the Blomberg's *K* statistic (Blomberg et al., 2003) and Pagel’s λ tests (Pagel, 1999) in the R package ‘phytools’. Larger values in *K* and λ indicate a greater phylogenetic conservatism for the given trait. Significance was testing *via* comparison of the variance of standardized contrasts to random values obtained by shuffling trait data across the tips of the tree 999 times.

To determine the effect of phylogenetic and environmental variables on the vein traits of all species, trees, and shrubs, the variance component of each trait was first partitioned into taxonomic, environmental (site), and residual components by using residual maximum likelihood procedures. The phylogenetic effect was defined as a hierarchically nested structure ‘(clade/order/family)’. The overall random term within the variance components model was (site + (clade/family/species)), and no fixed factors were defined. Thus, the variation in vein traits that was caused by environmental variables was assigned to the ‘site’ component of the model, whereas the variation that resulted from sampling error was assigned to the ‘residual’ term (Watanabe et al., 2007).

Subsequently, differences in vein traits from different altitudes were calculated, and each of the traits was analyzed using linear mixed-effects models with the restricted maximum likelihood method in the R package ‘lme4’. In these analyses, site was treated as a random effect, and the climatic variable, GF, and their interaction were treated as fixed effects. To avoid problems of collinearity among the climatic variables (Pearson's correlation coefficients >0.9), only the AI was included in these analyses. Due to the unbalanced data, the variance explained by the model was calculated using type III sums of squares and conservatively partitioned among fixed factors by calculating the variance explained by adding the focal factor after other factors had been included in the model. The sums of squares explained by random effect and its significance were estimated using the ‘r.squaredGLMM’ function and ‘exactRLRT' function in R package ‘MuMIn,’ respectively (Johnson, 2014).

## Results

### Trait Variation

**Figure 1:**
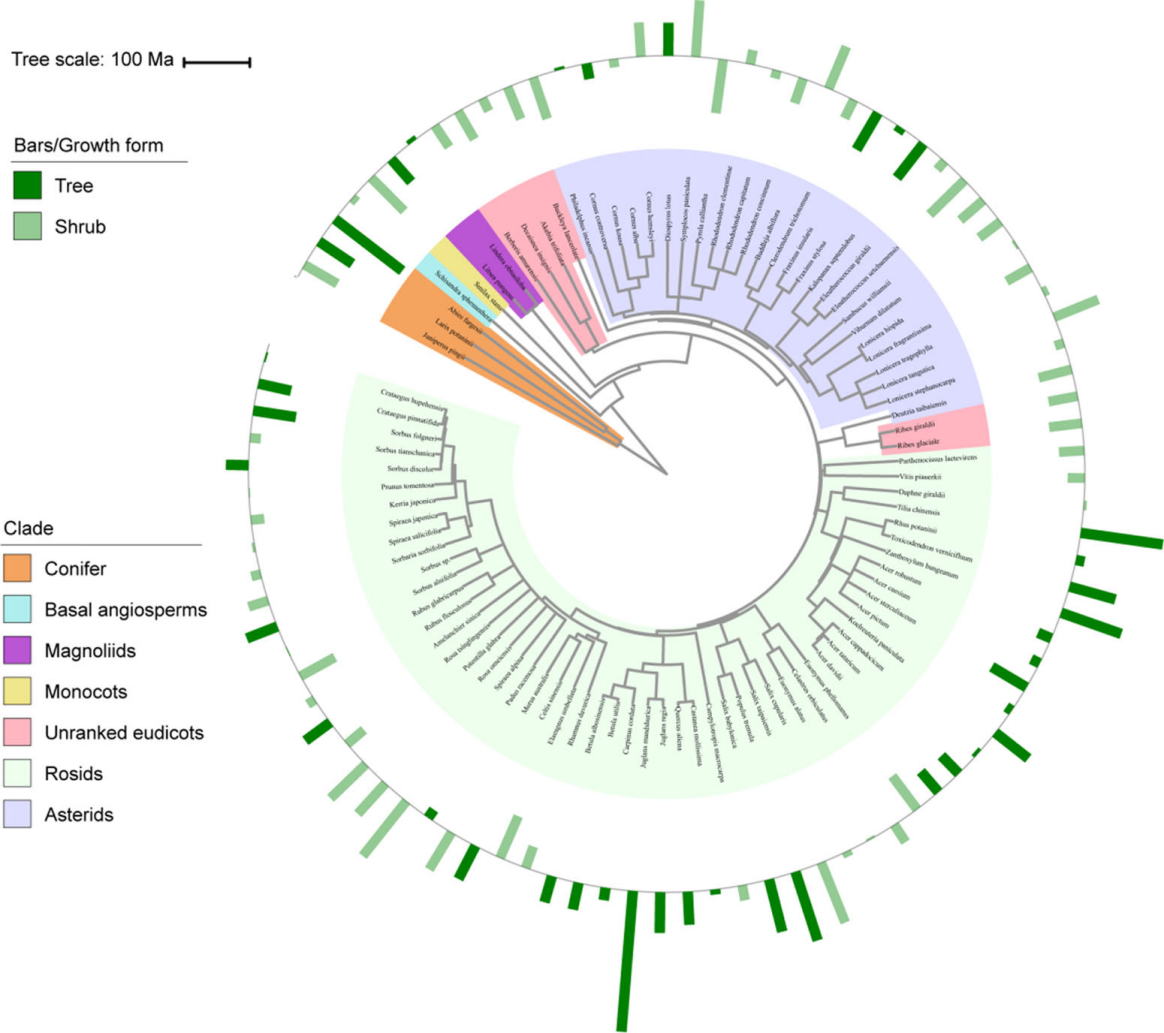
Variation in vein density (VD) with plant phylogeny and growth form. Bars represent standardized values for VD, with outward and inward bars representing values above and below the mean, respectively, and colors representing different growth forms. Branch colors in phylogeny represent main phylogenetic clades. Ma, million years ago.

Leaf vein traits of the 93 woody species varied greatly across species (**Table 1** and **Figure 1**). VD exhibited the least variation (coefficient of variation, CV = 0.34), ranging from 1.29 mm mm^–2^ in *A. fargesii* to 14.59 mm mm^–2^ in *Juglans mandshurica*, whereas VT exhibited inverse variation, ranging from 9.36 μm for *Castanea mollissima* to 182.74 μm for *A. fargesii*. Vein volume exhibited the greatest variation of the three measures (CV = 1.13).

**Table 1:** Differences in leaf vein traits among plant functional groups.

Plant functional group	*n*	VD (mm mm^-2^)			VT (μm)			VV (mm^3^ mm^-2^×10^-3^)	
		Mean ± SD	CV		Mean ± SD	CV		Mean ± SD	CV
All	93	6.34 ± 2.18	0.34		30.11 ± 21.30	0.71		5.00 ± 5.66	1.13
**Growth form**									
Tree	41	6.70 ± 2.49	0.37		35.05 ± 28.78	0.82		6.56 ± 7.40^a^	1.13
Shrub	52	6.05 ± 1.87	0.31		26.22 ± 11.58	0.44		3.76 ± 3.35^b^	0.89
*P* (*P_phy.__anova_*)		0.351 (0.331)			0.121 (0.334)			<0.001 (0.169)	
**Leaf type**									
Needled-leaves	3	3.10 ± 1.57^a^	0.51		113.02 ± 63.03^a^	0.56		26.72 ± 13.27^a^	0.50
Deciduous tree	38	6.99 ± 2.33^b^	0.33		28.89 ± 12.21^b^	0.42		4.97 ± 3.76^b^	0.76
Deciduous shrub	49	6.06 ± 1.91^b^	0.32		25.82 ± 11.72^b^	0.45		3.68 ± 3.41^b^	0.93
Evergreen shrub	3	5.96 ± 1.19^b^	0.20		32.75 ± 7.23^b^	0.22		5.15 ± 1.93^b^	0.38
*P* (*P_phy.__anova_*)		<0.001 (0.266)			<0.001 (0.115)			<0.001 (0.219)	

VD, vein density; VT, vein thickness; VV, vein volume per area; *n*, species number; CV, coefficient variation.

*P* and *P_phy.__anova_* denote the *P* values to test the effect of plant functional groups on the each vein trait according to standard and phylogenetic one-way analyses of variance (ANOVA).

Trait values are mean value ± 1 SD (standard deviation). Statistical differences are denoted by different letters (*P* < 0.05).

Remarkable trait variation also occurred among the plant functional groups (**Table 1** and **Figure 1**). Tree leaves tended to have greater VV than shrub leaves (*P <* 0.01) but similar VD and VT (both *P >* 0.05), and needled leaves had lower VD values than broad leaves but higher VT and VV (all *P <* 0.01), whereas there were no significant differences in any of the measures when comparing the evergreen and deciduous species (*P >* 0.05). Additionally, there were no significant differences when these data were examined by phylogenetic ANOVA.

In regard to the phylogenetic clades (**Figure 1** and **Supplementary Table S3**), different vascular plant groups populated distinctive ranges in vein traits. For example, conifers encompassed a narrow range of low VD values, ranging from 1.29 mm mm^–2^ in *A. fargesii* to 4.10 mm mm^–2^ in *Juniperus pingii*. Meanwhile, both the asterid and rosid species exhibited considerably broader ranges in VD, ranging from 3.14 to 9.7 mm mm^–2^ in the asterids and from 3.66 to 14.59 mm mm^–2^ in the rosids.

### Trait Correlation

**Figure 2:**
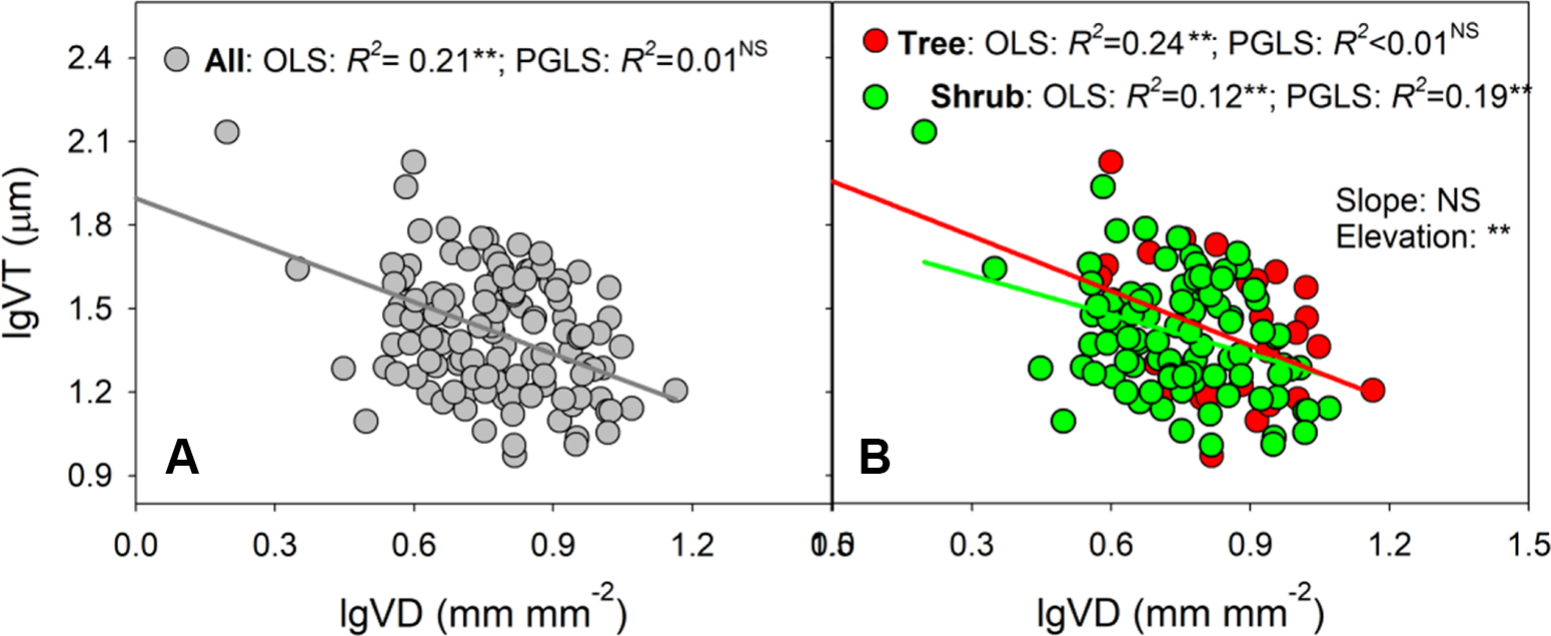
**(A, B)** Shows the relationships between vein thickness (VT) and density (VD) by ordinary least squares (OLS) and phylogenetic generalized least squares (PGLS) methods across all species, trees and shrubs, respectively. Model results of two methods are given in **Table S4**. In **(B)**, the differences in slopes and intercepts between trees and shrubs are tested *via* a standardized major axis (SMA) test (results given in **Table S5**). ‘Slope’, difference in standardized major axis (SMA) slopes; ‘Elevation’, difference in SMA elevations (y-intercept); NS, not significantly different; **P < 0.001.

Ordinary regression indicated strong negative relationships between VT and VD across all species, trees, and shrubs (*R^2^* values of OLS analyses = 0.12–0.24, all *P <* 0.01, **Figure 2** and **Supplementary Table S4**). In shrubs, VT remained significantly related to VD when phylogenetic effect was removed. However, the VT–VD relationship became insignificant after removing phylogenetic effect for all species (*R^2^* value of PGLS analysis = 0.01, *P >* 0.05) and trees (*R^2^* value of PGLS analysis <0.01, *P >* 0.05). In addition, the slope of the VT–VD relationship was affected little by growth form (*P* = 0.794), although the elevation (y-intercept) of the trees was clearly higher for shrubs (*P =* 0.008, **Supplementary Table S5**).

### Phylogenetic Patterns

Phylogenetic information exerted strong effects on leaf vein traits, but the strength of these effects was dependent on the trait measured and on plant growth form (**Table 2**). Across all species, only VD exhibited significant phylogenetic signals according to Blomberg's *K* (*K* = 0.38, *P <* 0.01), whereas the phylogenetic signals of VT and VV were significant according to the Pagel's test (*λ* = 0.74–0.82, all *P <* 0.01). For trees, all the vein traits exhibited significant phylogenetic signals according to both Blomberg's *K* and Pagel's λ values (value = 0.31–1.36, all *P <* 0.05). However, no significant phylogenetic signals were observed for the shrub species (value = < 0.01–0.37, all *P >*0.05).

**Table 2:** Blomberg's *K* and Pagel's λ values of leaf vein traits for different growth forms.

	All		Tree		Shrub	
	*K*	λ	*K*	λ	*K*	λ
VD	0.38^**^	0.74^**^	0.31^*^	0.76^*^	0.37	< 0.01
VT	1.03	0.82^**^	1.36^*^	0.90^**^	0.23	0.41
VV	0.77	0.78^**^	1.05^*^	0.86^**^	0.15	< 0.01

Trait abbreviations are provided in **Table 1**. *P <0.05; **P <0.01.

**Figure 3:**
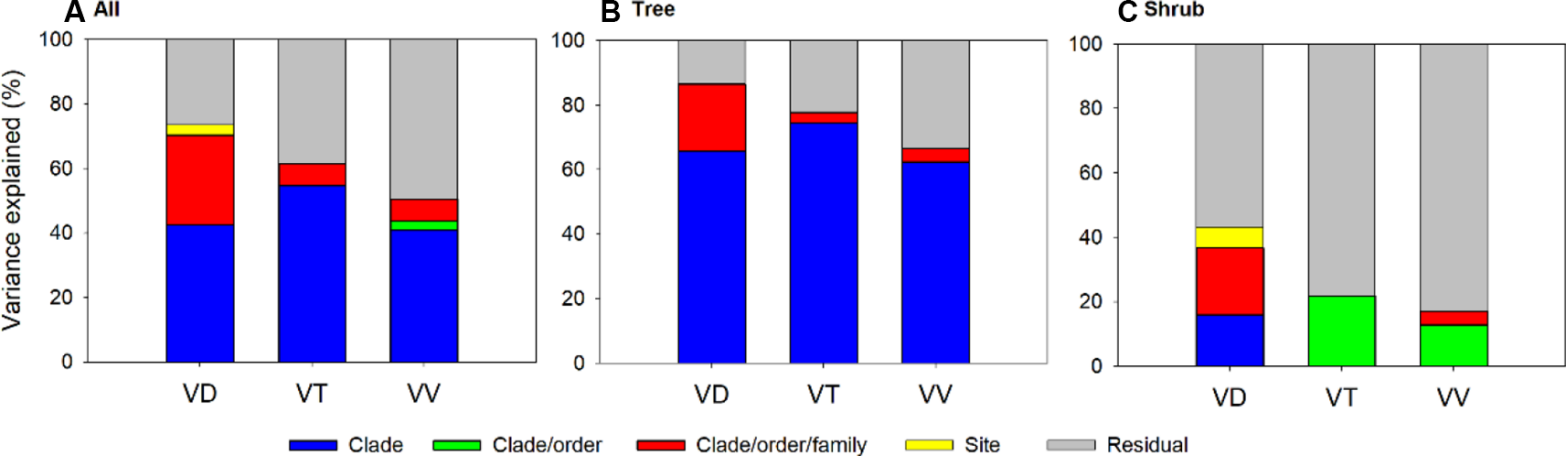
Variance component analysis of vein trains across all species **(A)**, trees **(B)** and shrubs **(C)** using phylogenetic nested analysis of variance (ANOVA). All traits are log_10_-transformed before analysis and their abbreviations are provided in **Table 1**.

Results of phylogenetically nested random model indicated that taxonomic effect was a considerable source of variation in the leaf vein traits (**Figure 3** and **Supplementary Table S6**). The variation explained by taxonomy (incorporating effects of clade, order and family) accounted for 17.1–86.6% of the total variation in the three traits, and the taxonomic effect was mainly observed at the clade level, reflecting substantial divergence between basal phylogenetic clades (e.g., gymnosperms and basal angiosperms) and the recently diverged clades (e.g., asterids and rosids). In addition, the influence of phylogenetic background was different between two growth forms, with more ‘clade’ effect detected for trees than shrubs (62.4–74.4% vs. <0.01–16.0%) and most of the variation in shrub traits left unexplained (>56%, **Supplementary Table S6**).

### Variation in Leaf Vein Traits Along Altitudinal Gradient

**Figure 4:**
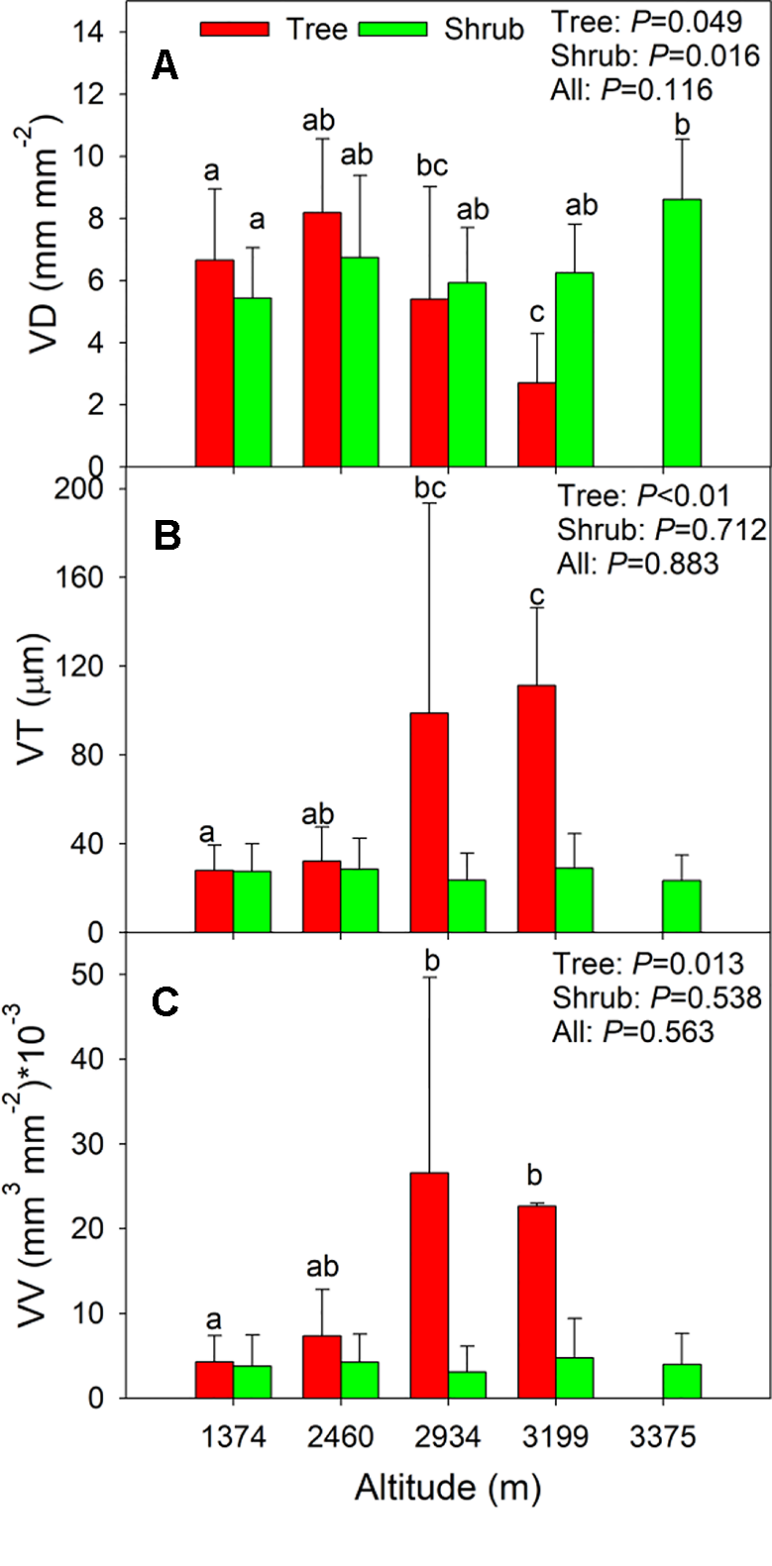
**(A–C)** Shows the changes in vein traits along the altitude for all species, trees, and shrubs. Error bars represent standard deviation. Different letters in each growth form indicate significant differences among altitudes (*P <*0.05). Trait abbreviations are provided in **Table 1**.

Across all species, there were no clear altitudinal trends in the three leaf vein traits (*P =* 0.116–0.883, **Figure 4**), and different altitude-related changes were observed for the trees and shrubs. In trees, plants from higher altitudes (i.e., 2,934 m and 3,199 m, **Supplementary Table S1**) possessed lower VD but higher VT and VV than those from lower altitudes (*P = <* 0.01–0.049, **Figure 4**). In contrast, the VD of shrubs from the alpine shrubland (3,375 m) was significantly higher than that of shrubs from the *Quercus* forest (1374 m, *P =* 0.016, **Figure 4A**). There were no significant changes in VT or VV of shrub species along the altitudinal gradients (*P =* 0.712–0.538, **Figures 4B, C**).

**Figure 5:**
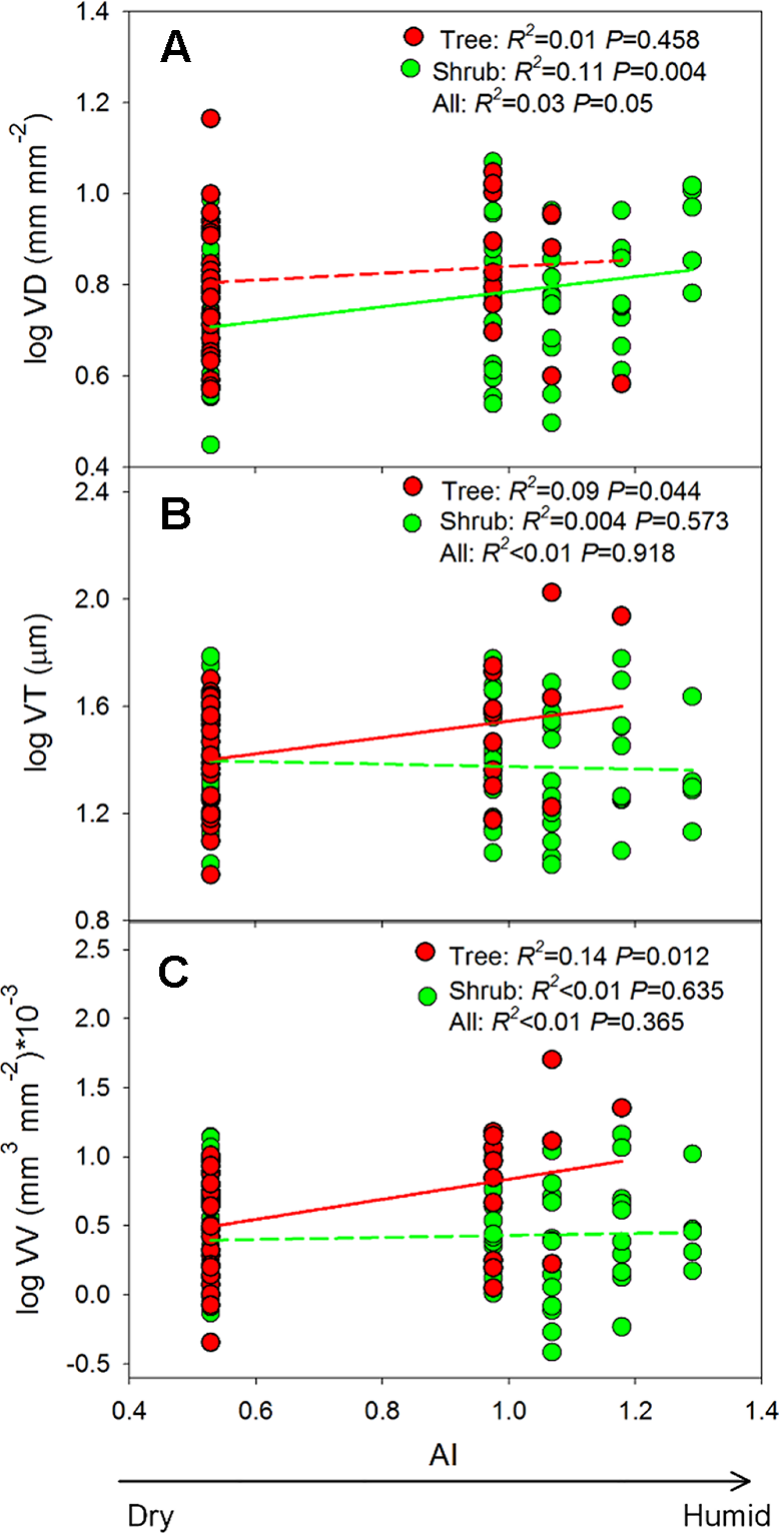
**(A**–**C)** Shows the changes in vein traits along the aridity gradient. AI, aridity index. Insignificant regression lines are shown in dotted lines (*P >* 0.05). Trait abbreviations are provided in **Table 1**.

Mixed-effect models, which were used to quantify the effects of growth form and aridity index (AI) on root trait variation, indicated that both fixed and random factors (i.e., *R^2^*_c_) could explain 15–21% of the variation in VD, VT, and VV (**Table 3**). Growth form and its interaction with AI significantly affected altitudinal changes in the leaf vein traits (all *P <*0.01), explaining 1.11–9.95% of the total variance (**Table 3**). For trees, both VT and VV increased with increasing AI (*P* = 0.012–0.044, **Figures 5B, C**), whereas VD did not (*P* = 0.458, **Figure 5A**), and in shrubs, only VD increased significantly with increasing AI (*P* = 0.004, **Figure 5A**). It should be emphasized, however, that the *R^2^*-values of these relationships were all below 0.15 (**Figure 5**), indicating the relatively weak effect of climate.

**Table 3:** Statistical model for altitude-related variation in vein trait as a function of aridity index (AI), growth form (GF), and their interaction.

Fixed Effect	*df*	VD				VT				VV			
		Estimate	Std.Error	*P*	SS%	Estimate	Std.Error	*P*	SS%	Estimate	Std.Error	*P*	SS%
Intercept	118	0.63	0.14	0.036		1.43	0.08	< 0.001		0.39	0.15	0.009	
GF	118	0.28	0.09	0.003	0.47	−0.31	0.13	0.018	4.00	-0.38	0.23	0.105	6.39
AI	118	0.16	0.14	0.339	1.12	−0.05	0.09	0.571	0.53	0.05	0.16	0.764	1.22
GF×AI	118	−0.35	0.12	0.005	1.11	0.57	0.16	0.001	9.95	0.86	0.29	0.004	12.47
*R^2^*_m_		0.06				0.15				0.15			
*R^2^*_c_		0.21				0.15				0.15			

df, degree of freedom; SS%, percentage of sum of squares explained. All the trait data are log_10_-transformed prior to analysis and their abbreviations are in **Table 1**.

Linear mixed-effects model was fit by restricted maximum likelihood. Random effects in model were ‘site’. Marginal R^2^ (R^2^_m_) is concerned with variance explained by fixed factors, and conditional R^2^ (R^2^_c_) is concerned with variance explained by both fixed and random factors.

## Discussion

Based on a large dataset, four key findings were generated by the present study. First, when all species were considered, no clear trends were observed for the vein traits along the altitudinal gradient, and phylogenetic effect explained most of the variation at the clade level, whereas climate exerted very weak influence. Second, the altitude-related patterns in the vein traits of the trees and shrubs differed significantly. In trees, plants from high altitude tended to produce the leaves with the relatively lower VD than those of low altitude. However, the opposite trend was observed in shrubs. Third, the effect of phylogeny on the vein traits differed between trees and shrubs, with tree vein traits being relatively more conserved. Finally, a trade-off between VT and VD was observed for all species, trees, and shrubs, but this relationship was dependent on phylogeny. Overall, our results succeed in quantifying the effects of plant phylogeny, growth form, and environmental variables on leaf vein traits, and provide strong support for the leaf vein trait phylogenetic conservatism hypothesis.

### Effects of Plant Phylogeney on Leaf Vein Traits

In the present study, both Pagel's λ test and the nested random model supported the hypothesis that phylogeny exerted a strong effect on species-level variation in leaf vein traits. Even VD, which is generally considered quite labile (Uhl and Mosbrugger, 1999; Sack and Scoffoni, 2013), had significant phylogenetic signal at this scale, consistent with the data of some woody plant lineages (Zhang et al., 2012; Liu et al., 2015). However, a previous study of 130 woody angiosperm species reported a lack of phylogenetic signal for vein traits, as evidenced by low Blomberg's *K* values and non-zero phylogenetic regression slopes (Blonder et al., 2017). This inconsistency could be attributed to the use of different phylogenetic signal indices and tests. Münkemüller et al. (2012) proposed that Pagel's λ generally outperformed *K* in detecting phylogenetic signals, whereas *K* was suitable for models with changing evolutionary rates. Indeed, in the present study, both Blomberg's *K* and Pagel's λ tests were used. Yet, only the Pagel's λ tests indicated that there were phylogenetic effects on VT and VV for all species. Another explanation for this inconsistence is that Blonder et al. (2017) only sampled the dominant angiosperms of the forest communities and, thereby, may limited the range of phylogenetic lineages.

In the present study, both the strong conservatism of vein traits and most of the trait variation occurred among different clades, which suggests that leaf venation networks have evolved slowly since the major phylogenetic groups have come into being. Specifically, species from early clades (e.g., conifers) tended to possess low VD but high VT and VV, whereas species from recently diverged clades (e.g., asterid and rosid) tended to possess low VT, with relatively high VD (**Figure 1** and **Supplementary Table S3**). Furthermore, VD values of ≥10 mm mm^–2^ were only found in the rosid species. Similar evidence can be found in the dataset compiled from both freshly collected leaves and herbarium materials (Boyce et al., 2009), which indicated that angiosperm VD ranges from >5 mm mm^–2^ to 25 mm mm^–2^, whereas non-angiosperm VD consistently averages around 2 mm mm^–2^. Indeed, the high VD observed across modern angiosperms is thought to confer a greater capacity for photosynthesis and has been interpreted as a critical adaptation of angiosperms to dominate in productive environments (Brodribb and Feild, 2010; Brodribb et al., 2010). Taken together, these results provide strong support for the leaf vein trait phylogenetic conservatism hypothesis.

In addition, the effects of phylogeny on the leaf vein traits were different for the trees and shrubs. For example, the overall phylogenetic structure, at the clade level, explained more variation in the leaf vein traits of tree species than that of shrub species (**Figure 3** and **Supplementary Tables S5**), and no significant phylogenetic signals were found for the shrub vein traits (**Table 1**). In general, phylogenetic signals can be caused by different processes. Strong phylogenetic signals can result from a variety of evolutionary processes, such as phylogenetic inertia, stabilizing selection, and evolution by Brownian motion among related species, whereas weak signals might indicate evolutionary labile or adaptation to environmental conditions through natural selection (Blomberg and Garland, 2002; Crisp and Cook, 2012). Thus, the lack of a phylogenetic signal in these variables indicates that the leaf venation of shrubs might be affected more by environmental change than by differences among species. Indeed, it is generally acknowledged that in compared with trees, shrubs often have high adaptation to environmental changes and unfavorable habitats (Woodward, 1993; Niinemets, 2001). For example, maximum plant stature decreases with increasing drought stress (Woodward, 1993), and tree species with dense and costly evergreen leaves are replaced by drought-deciduous shrubs and ephemeral life-forms, which are active only during short periods of ample water availability (Woodward, 1993; Niinemets, 2001). Similarly, trees cannot continue to grow when altitude reaches at a certain height (i.e., treeline), and only shrubs and herbaceous plants can survive and dominate in high altitudes (**Supplementary Figure S1**).

### Effect of Climate on Leaf Vein Traits

Identifying the relationships between leaf vein traits and the environment is helpful for understanding the distribution of plant species across environments and for paleoclimate reconstruction (Uhl and Mosbrugger, 1999; Sack and Scoffoni, 2013; Blonder and Enquist, 2014; Blonder et al., 2017). However, the ways in which vein traits change along environmental gradients are not fully understood. In previous studies, the trend of increasing VD at lower altitudes has been reported both across multiple tree species (Zhao et al., 2016) and at the community scale (Blonder et al., 2017). However, in the present study, no clear altitude-related trends in leaf vein traits were observed across all species, and the observed patterns varied among the different growth forms (**Table 3** and **Figure 5**).

Considerable variations were observed between growth forms, which reflected differences in the adaptive strategies of trees and shrubs to their external environment. In Taibai Mountain, deciduous trees with thin but dense leaf vein networks were dominant at lower altitudes (e.g., *Quercus* spp.), whereas conifers, which had contrasting characters, generally dominated at higher altitudes (e.g., *A. fargesii* and *L. potaninii* var. *chinensis*) (**Supplementary Tables S1**). Therefore, shifts in dominant species may have contributed to the observed trends of lower VD but greater VT and VV in trees at high altitudes (**Figure 4**). For shrubs, VD increased with increasing altitude and peaked at 3,375 m (i.e., alpine shrubland). In previous studies, plants grown at high altitudes possessed both high stomatal density and leaf nitrogen concentrations (Körner, 1989; Wang et al., 2014). The high VD of shrubs at high altitudes may be matched with the high stomatal conductance (determined by stomatal density) and photosynthetic capacity (associated with leaf nitrogen concentration) during periods of higher resource availability, in order to compensate for the short growth period. Therefore, shifts in species composition along environmental gradients contributed substantially to generating the observed variations in vein traits.

Water availability is often regarded as an important factor that affects the variation of leaf vein networks. For example, the negative relationship between VD and water availability has been clearly observed across biomes at the global scale (Sack and Scoffoni, 2013). However, positive correlations were observed in our study (**Figure 5**), and despite significant relationships between traits and the aridity index, the *R^2^* values of these relationships were all below 0.15 (**Figure 5**). The weakness of these correlations can mainly be attributed to the observation that a substantial proportion of the trait variation occurred at the within-site level and, thus, could not be explained by differences in the climate of the study sites (**Figure 3**) (also found in Blonder and Enquist, 2014). Within communities, plants can use different strategies to adapt to external environments, thereby masking predicted relationships (Schneider et al., 2018). For example, small and waxy leaves with stiff leaf cell walls, which are common in shrubs of high altitudes, indicate high investments in avoiding injury by unfavorable environments (Milla and Reich, 2011). Differently, another species dominant at high altitudes (i.e., *L. potaninii* var. *chinensis*, **Supplementary Table S1**) sheds its needles as an adaptive strategy to environmental stress.

Furthermore, changes in altitude are often associated with changes in a variety of environmental factors, thereby obscuring the effect of single factors (Körner, 2007). For example, the low air temperatures that occur at high altitudes could reduce transpiration demand and the flow rate of the venation system (Brodribb et al., 2010; Sack and Scoffoni, 2013), whereas both the high wind velocity and high UV-B radiation could increase the transpiration rate of leaves (Körner, 2007).

### Trade-Off Between Vein Thickness and Density

Consistent with the general trade-off between VT and VD reported by previous studies (Sack et al., 2012; Feild and Brodribb, 2013), the present study revealed that VT was negatively correlated with VD across all species and growth forms; however, these relationships were dependent on phylogenetic background (**Figure 2** and **Supplementary Table S4**). For example, when phylogenetic analyses were conducted, the observed trade-off only remained significant in shrubs. For trees and all species, the VT–VD relationships were insignificant, possibly due to the conservatism of trait combinations in relatively few clades, rather than correlated evolution (Felsenstein, 1985; Walls, 2011).

The anatomic structure and tissue displacement inside leaf vein networks influence the balance between CO_2_ uptake for plant photosynthesis and water loss by transpiration (Boyce et al., 2009; Beerling and Franks, 2010; Feild and Brodribb, 2013; Sack and Scoffoni, 2013).The trade-off between VT and VD can be partially explained by physical space constraints, cost-benefit theory, and evolutionary coevolution (Feild and Brodribb, 2013). The physical space constraints hypothesis assumes that, like stomata, there must be a minimum allowable proximity between elements of minor veins (Franks et al., 2009; Franks et al., 2012). The distance of veins to one another is constrained by the requirement of a minimal CO_2_ diffusional continuum between the stomata and photosynthetic tissue (Brodribb et al., 2007). If veins became so widely or densely packed as to coalesce, the vertical connection between the stomata and the photosynthetic tissue would be cut off, since xylem is impermeable to CO_2_ diffusion (Brodribb et al., 2007; Feild and Brodribb, 2013; Blonder et al., 2017). Because, veins occupy a single plane in leaves, the only way to increase VD without intersecting neighboring veins is to reduce VT (Feild and Brodribb, 2013).

High VD can enable higher leaf hydraulic conductance, greater stomatal conductance, and higher rates of gas exchange per leaf area (Brodribb et al., 2007; Brodribb and Feild, 2010; Sack et al., 2012; Sack and Scoffoni, 2013; McElwain et al., 2016). However, high VD also implies high leaf-construction costs because it requires additional specialized water-conducting tissues, which are rich in carbon-costly lignin (Beerling and Franks, 2010; McKown et al., 2010). From the perspective of cost-benefit theory, increasing the density of minor veins together with vein tapering is, by far, the most cost-effective strategy, since it allows higher leaf hydraulic conductance and faster rates of photosynthesis for a given carbon investment in lignified tissues (Beerling and Franks, 2010; McKown et al., 2010). This innovation has far-reaching implications for evolutionary innovations in angiosperms. This advanced leaf venation, together with a more sophisticated stomatal control system and thinner root structures, set the stage for the rise of angiosperms to global dominance following the early Cretaceous (130 million years ago), in response to falling atmospheric CO_2_ concentrations (Beerling and Franks, 2010; Comas et al., 2012; Sack and Scoffoni, 2013).

Another mechanism that could responsible for the observed reductions in vein conduit size is the evolutionary down-sizing of genome size (Feild and Brodribb, 2013). With all else being equal, reducing genome size is generally associated with decreasing cell size (Franks et al., 2012). For example, in angiosperms, genome size is a strong and positive predictor of guard cell length (Franks et al., 2012). Thus, to maintain a balance between hydraulic supply (associated with leaf hydraulic conductance) and transpirational loss (related to stomatal conductance), it is clear that structural assembly of terrestrial angiosperm's ability to make productive leaves involved small, but dense, stomata and minor veins (Franks et al., 2012; Feild and Brodribb, 2013; Sack et al., 2013).

## Conclusion

In this study, we tested the leaf vein trait phylogenetic conservatism hypothesis using 93 woody species along an altitudinal gradient. As expected, we found that plant phylogeny exerted an important influence on the variation of leaf vein traits, whereas climate had a relatively weak effect. In addition, the altitude-related trends of leaf vein traits differed between growth forms, which could partially explain the inconsistent conclusion pertaining to trait–climate relationships found in previous studies. Given that environmental differences between sites have relatively little influence on species-level trait variation, the use of community-aggregated values would be a promising strategy for identifying trait-environment relationships and for predicting them on a quantitatively basis (Violle et al., 2014; Blonder et al., 2017). Finally, because the present study sampled a limited number of species (and families), especially for early-divergent groups, evolutionary mechanisms underlying vein trait variation were not well understood. Therefore, in order to enhance our understanding of leaf vein trait evolution, a greater number of early-diverging groups, such as conifers and basal angiosperms, should be included in future studies.

## Data Availability Statement

All datasets generated for this study are included in the article/**Supplementary Material**.

## Author Contributions

RW and SZ conceived the ideas and designed the methodology. HC, XL, and JW collected the data. RW and ZW analyzed the data. RW wrote the manuscript.

## Funding

This work was supported by the National Natural Science Foundation of China (31700381), the National Key R&D Program of China (2016YFC0500202, 2017YFA0604803), China Postdoctoral Science Foundation (2017M623252, 2018T111101).

## Conflict of Interest

The authors declare that the research was conducted in the absence of any commercial or financial relationships that could be construed as a potential conflict of interest.

## References

[B1] APG (The Angiosperm Phylogeny Group) (2016). An update of the Angiosperm Phylogeny Group classification for the orders and families of flowering plants: APG IV. Bot. J. Linn. Soc. 181, 1–20. 10.1111/boj.12385

[B2] Beerling D. J. Franks P. J. (2010). The hidden cost of transpiration. Nature 464 (7288), 495–496. 10.1038/464495a 20336123

[B3] Blomberg S. P. Garland T. (2002). Tempo and mode in evolution: phylogenetic inertia, adaptation and comparative methods. J. Evol. Biol. 15 (6), 899–910. 10.1046/j.1420-9101.2002.00472.x

[B4] Blomberg S. P. Garland T. Ives A. R. (2003). Testing for phylogenetic signal in comparative data: behavioral traits are more labile. Evolution 57 (4), 717–745. 10.1111/j.0014-3820.2003.tb00285.x 12778543

[B5] Blonder B. Enquist B. J. (2014). Inferring climate from angiosperm leaf venation networks. New Phytol. 204 (1), 116–126. 10.1111/nph.12780 24725225

[B6] Blonder B. Salinas N. Bentley L. P. Shenkin A. Porroa P. O. C. Tejeira Y. V. (2017). Predicting trait-environment relationships for venation networks along an Andes-Amazon elevation gradient. Ecology 98 (5), 1239–1255. 10.1002/ecy.1747 28122124

[B7] Boyce C. K. Brodribb T. J. Feild T. S. Zwieniecki M. A. (2009). Angiosperm leaf vein evolution was physiologically and environmentally transformative. P. R. Soc. B-Biol. Sci. 276 (1663), 1771–1776. 10.1098/rspb.2008.1919 PMC2674498 19324775

[B8] Brodribb T. J. Feild T. S. (2010). Leaf hydraulic evolution led a surge in leaf photosynthetic capacity during early angiosperm diversification. Ecol. Lett. 13 (2), 175–183. 10.1111/j.1461-0248.2009.01410.x 19968696

[B9] Brodribb T. J. Feild T. S. Jordan G. J. (2007). Leaf maximum photosynthetic rate and venation are linked by hydraulics. Plant Physiol. 144 (4), 1890–1898. 10.1104/pp.107.101352 17556506 PMC1949879

[B10] Brodribb T. J. Feild T. S. Sack L. (2010). Viewing leaf structure and evolution from a hydraulic perspective. Funct. Plant Biol. 37 (6), 488–498. 10.1071/FP10010

[B11] Comas L. Mueller K. Taylor L. Midford P. Callahan H. Beerling D. (2012). Evolutionary patterns and biogeochemical significance of angiosperm root traits. Int. J. Plant Sci. 173 (6), 584–595. 10.1086/665823

[B12] Crisp M. D. Cook L. G. (2012). Phylogenetic niche conservatism: what are the underlying evolutionary and ecological causes? New Phytol. 196 (3), 681–694. 10.1111/j.1469-8137.2012.04298.x 22943495

[B13] de Boer H. J. Eppinga M. B. Wassen M. J. Dekker S. C. (2012). A critical transition in leaf evolution facilitated the Cretaceous angiosperm revolution. Nat. Commun. 3, 1221. 10.1038/ncomms2217 23187621 PMC3514505

[B14] Feild T. S. Brodribb T. J. (2013). Hydraulic tuning of vein cell microstructure in the evolution of angiosperm venation networks. New Phytol. 199 (3), 720–726. 10.1111/nph.12311 23668223

[B15] Feild T. S. Chatelet D. S. Brodribb T. J. (2009). Ancestral xerophobia: a hypothesis on the whole plant ecophysiology of early angiosperms. Geobiology 7 (2), 237–264. 10.1111/j.1472-4669.2009.00189.x 19260972

[B16] Felsenstein J. (1985). Phylogenies and the comparative method. Am. Nat. 125 (1), 1–15.

[B17] Franks P. J. Drake P. L. Beerling D. J. (2009). Plasticity in maximum stomatal conductance constrained by negative correlation between stomatal size and density: an analysis using *Eucalyptus globulus* . Plant Cell Environ. 32 (12), 1737–1748. 10.1111/j.1365-3040.2009.002031.x 19682293

[B18] Franks P. J. Freckleton R. P. Beaulieu J. M. Leitch I. J. Beerling D. J. (2012). Megacycles of atmospheric carbon dioxide concentration correlate with fossil plant genome size. Phil. Trans. R. Soc. B 367 (1588), 556–564. 10.1098/rstb.2011.0269 22232767 PMC3248711

[B19] Griffin-Nolan R. J. Bushey J. A. Carroll C. J. W. Challis A. Chieppa J. Garbowski M. (2018). Trait selection and community weighting are key to understanding ecosystem responses to changing precipitation regimes. Funct. Ecol. 32 (7), 1746–1756. 10.1111/1365-2435.13135

[B20] Johnson P. C. (2014). Extension of Nakagawa & Schielzeth's *R^2^* _GLMM_ to random slopes models. Methods Ecol. Evol. 5 (9), 944–946. 10.1111/2041-210X.12225 25810896 PMC4368045

[B21] Körner C. (1989). The nutritional status of plants from high altitudes: a worldwide comparison. Oecologia 81 (3), 379–391. 10.1007/BF00377088 28311193

[B22] Körner C. (2007). The use of ‘altitude' in ecological research. Trends Ecol. Evol. 22 (11), 569–574. 10.1016/j.tree.2007.09.006 17988759

[B23] Liu H. Xu Q. He P. Santiago L. S. Yang K. Ye Q. (2015). Strong phylogenetic signals and phylogenetic niche conservatism in ecophysiological traits across divergent lineages of Magnoliaceae. Sci. Rep. 5, 12246. 10.1038/srep12246 26179320 PMC4503962

[B24] Münkemüller T. Lavergne S. Bzeznik B. Dray S. Jombart T. Schiffers K. (2012). How to measure and test phylogenetic signal. Methods Ecol. Evol. 3 (4), 743–756. 10.1111/j.2041-210X.2012.00196.x

[B25] McElwain J. C. Yiotis C. Lawson T. (2016). Using modern plant trait relationships between observed and theoretical maximum stomatal conductance and vein density to examine patterns of plant macroevolution. New Phytol. 209 (1), 94–103. 10.1111/nph.13579 26230251 PMC5014202

[B26] McGill B. J. Enquist B. J. Weiher E. Westoby M. (2006). Rebuilding community ecology from functional traits. Trends Ecol. Evol. 21 (4), 178–185. 10.1016/j.tree.2006.02.002 16701083

[B27] McKown A. D. Cochard H. Sack L. (2010). Decoding leaf hydraulics with a spatially explicit model: principles of venation architecture and implications for its evolution. Am. Nat. 175 (4), 447–460. 10.1086/650721 20178410

[B28] Milla R. N. Reich P. B. (2011). Multi-trait interactions, not phylogeny, fine-tune leaf size reduction with increasing altitude. Ann. Bot-London 107, 455–465. 10.1093/aob/mcq261 PMC3043936 21199835

[B29] Niinemets Ü. (2001). Global-scale climatic controls of leaf dry mass per area, density, and thickness in trees and shrubs. Ecology 82 (2), 453–469. 10.1890/0012-9658(2001)082[0453:GSCCOL]2.0.CO;2

[B30] Pagel M. (1999). Inferring the historical patterns of biological evolution. Nature 401 (6756), 877–884. 10.1038/44766 10553904

[B31] Ren Y. Liu M. Tian L. Li Z. (2006). Biodiversity, conservation and management of Taibaishan Nature Reserve (Beijing: Chinese Foresty Press).

[B32] Sack L. Scoffoni C. (2013). Leaf venation: structure, function, development, evolution, ecology and applications in the past, present and future. New Phytol. 198 (4), 983–1000. 10.1111/nph.12253 23600478

[B33] Sack L. Scoffoni C. McKown A. D. Frole K. Rawls M. Havran J. C. (2012). Developmentally based scaling of leaf venation architecture explains global ecological patterns. Nat. Commun. 3, 837. 10.1038/ncomms1835 22588299

[B34] Sack L. Scoffoni C. John G. P. Poorter H. Mason C. M. Mendez-Alonzo R. (2013). How do leaf veins influence the worldwide leaf economic spectrum? Review and synthesis. J. Exp. Bot. 64 (13), 4053–4080. 10.1093/jxb/ert316 24123455

[B35] Schneider J. V. Negraschis V. Habersetzer J. Rabenstein R. Wesenberg J. Wesche K. (2018). Taxonomic diversity masks leaf vein-climate relationships: lessons from herbarium collections across a latitudinal rainfall gradient in West Africa. Bot. Lett. 165 (3-4), 384–395. 10.1080/23818107.2017.1421480

[B36] Tang Z. Y. Fang J. Y. (2006). Temperature variation along the northern and southern slopes of Mt. Taibai, China. Agr. For. Meteorol. 139 (3-4), 200–207. 10.1016/j.agrformet.2006.07.001

[B37] Uhl D. Mosbrugger V. (1999). Leaf venation density as a climate and environmental proxy: a critical review and new data. Palaeogeogr. Palaeocl. 149 (1-4), 15–26. 10.1016/S0031-0182(98)00189-8

[B38] Violle C. Reich P. B. Pacala S. W. Enquist B. J. Kattge J. (2014). The emergence and promise of functional biogeography. P. Natl. Acad. Sci. U.S.A. 111 (38), 13690–13696. 10.1073/pnas.1415442111 PMC4183284 25225414

[B39] Walls R. L. (2011). Angiosperm leaf vein patterns are linked to leaf functions in a global-scale data set. Am. J. Bot. 98 (2), 244–253. 10.3732/ajb.1000154 21613113

[B40] Wang R. L. Yu G. R. He N. P. Wang Q. F. Xia F. C. Zhao N. (2014). Elevation-related variation in leaf stomatal traits as a function of plant functional type: evidence from Changbai Mountain, China. PloS One 9 (12), e115395. 10.111371/journal.pone.0115395 25517967 PMC4269444

[B41] Watanabe T. Broadley M. R. Jansen S. White P. J. Takada J. Satake K. (2007). Evolutionary control of leaf element composition in plants. New Phytol. 174 (3), 516–523. 10.1111/j.1469-8137.2007.02078.x 17447908

[B42] Woodward F. I. (1993). “Leaf responses to the environment and extrapolation to larger scales,” in Vegetation dynamics & global chang. Eds. Solomon A. M. Shugart H. H. (New York: Chapman & Hall Press), 71–100.

[B43] Zanne A. E. Tank D. C. Cornwell W. K. Eastman J. M. Smith S. A. FitzJohn R. G. (2014). Three keys to the radiation of angiosperms into freezing environments. Nature 506 (7486), 89–92. 10.1038/nature12872 24362564

[B44] Zhang S. B. Guan Z. J. Sun M. Zhang J. J. Cao K. F. Hu H. (2012). Evolutionary association of stomatal traits with leaf vein density in *Paphiopedilum*, Orchidaceae. PloS One 7 (6), e40080. 10.1371/journal.pone.0040080 22768224 PMC3386932

[B45] Zhao W. L. Chen Y. J. Brodribb T. J. Cao K. F. (2016). Weak co-ordination between vein and stomatal densities in 105 angiosperm tree species along altitudinal gradients in Southwest China. Funct. Plant Biol. 43 (12), 1126–1133. 10.1071/FP16012 32480532

