# Supplementary material for: Plant Phylogeny and Growth Form as Drivers of the Altitudinal Variation in Woody Leaf Vein Traits
Source: Front Plant Sci. 2020 Feb 5;10:1735. doi: 10.3389/fpls.2019.01735 (PMC7012802; doi:10.3389/fpls.2019.01735)
Supplement: Supplementary file 1 [file DataSheet_1.docx]

# Tables

**Supplementary Table S1.** Characteristics of five sampling sites along an altitudinal gradient on Taibai Mountain**,** China.

| Altitude (m a.s.l.) | Latitude  (°N) | Longitude  (°E) | MAT  (°C) | MAP (mm) | Vegetation type | Dominant species |
| --- | --- | --- | --- | --- | --- | --- |
| 1374 | 34.26 | 108.06 | 11.17 | 656 | Deciduous broadleaf forest | *Quercus aliena* var. *acuteserrata* |
| 2460 | 34.01 | 107.82 | 5.95 | 793 | Birch forest | *Betula albosinensis*  *B. utilis* |
| 2934 | 34.01 | 107.81 | 3.53 | 806 | Fir forest | *Abies fargesii* |
| 3199 | 34.00 | 107.81 | 2.32 | 840 | Larch forest | *Larix potaninii* var. *chinensis* |
| 3375 | 33.99 | 107.80 | 1.36 | 863 | Alpine shrubland | *Rhododendron capitatum* |

MAP, mean annual precipitation; MAT, mean annual temperature. MAP and MAT are calculated according to empirical equations (details in main text).

**Supplementary Table S2.** Species number of plant functional types and major clades sampled at each site on the Taibai Mountain.

| Altitude  (m a.s.l.) | Tree | |  | Shrub | | All |
| --- | --- | --- | --- | --- | --- | --- |
|  | Conifer | Deciduous |  | Deciduous | Evergreen |  |
| 1374 |  | 32 |  | 31 |  | 63 |
| 2460 | 1 | 9 |  | 16 |  | 26 |
| 2934 | 2 | 2 |  | 11 | 2 | 17 |
| 3199 | 3 |  |  | 7 | 2 | 12 |
| 3375 |  |  |  | 4 | 1 | 5 |

Plant functional type included conifer and woody angiosperms (deciduous tree, deciduous shrub, and evergreen shrub).

Notably, all conifers except *Larix potaninii* var. *chinensis* are evergreen trees. All of five evergreen angiosperm plants belong to Ericaceae family and shrub.

| Altitude (m) | Conifer | Basal angiosperms | Magnoliids | Monocots | Unranked eudicots^†^ | Rosids | Asterids |
| --- | --- | --- | --- | --- | --- | --- | --- |
| 1374 |  | 1 | 2 | 1 | 4 | 40 | 15 |
| 2460 | 1 |  |  | 1 | 2 | 15 | 7 |
| 2934 | 2 |  |  |  | 2 | 7 | 6 |
| 3199 | 3 |  |  |  | 1 | 3 | 5 |
| 3375 |  |  |  |  |  | 3 | 2 |

^†^, Angiosperm order and family assignments are based on the Angiosperm Phylogeny Group IV classification. Unranked eudicots species are those not included in the clades of rosids and asterids.

**Supplementary Table S3.** Differences in leaf vein traits among phylogenetic clades.

| Clade | *n* | VD | |  | VT | |  | VV | |
| --- | --- | --- | --- | --- | --- | --- | --- | --- | --- |
|  |  | Mean ± SD | CV |  | Mean ± SD | CV |  | Mean ± SD | CV |
| Conifer | 3 | 3.09 ± 1.57^a^ | 0.51 |  | 113.02 ±63.03^a^ | 0.56 |  | 26.72 ±13.27^a^ | 0.50 |
| Basal angiosperms | 1 | 5.99 |  |  | 30.86 |  |  | 4.48 |  |
| Magnoliids | 2 | 5.50 ± 1.43 | 0.26 |  | 29.86 ± 7.75 | 0.26 |  | 4.24 ± 3.02 | 0.71 |
| Monocots | 1 | 3.58 |  |  | 45.45 |  |  | 5.80 |  |
| Unranked eudicots^†^ | 6 | 4.68 ± 0.45^a^ | 0.10 |  | 24.82 ± 9.61^b^ | 0.39 |  | 2.65 ± 2.34^b^ | 0.88 |
| Rosids | 54 | 7.04 ± 2.18^b^ | 0.31 |  | 27.93 ± 12.48b | 0.45 |  | 4.87 ± 3.94^b^ | 0.81 |
| Asterids | 26 | 5.83 ± 1.85^a^ | 0.35 |  | 25.70 ± 11.39^b^ | 0.44 |  | 3.35 ± 2.90^b^ | 0.87 |
| *P* (*P_phy. anova_*) ^‡^ |  | <0.001 (0.228) | |  | 0.032 (0.231) | |  | 0.011(0.418) | |

VD, vein density; VT, vein thickness; VV, vein volume per area; *n*, species number.

*P* and *P_phy. anova_* denote the *P* values to test the effect of plant phylogenetic calde on the each vein trait according to standard and phylogenetic one-way analyses of variance (ANOVA). Angiosperm order and family assignments are based on the Angiosperm Phylogeny Group IV classification. Trait values are mean value ± 1 SD (standard deviation). Statistical differences are denoted by different letters (*P* < 0.05).

^†^, Unranked eudicots species are those not included in the clades of rosids and asterids.

^‡^, Species of basal angiosperms, magnoliids, and monocots were excluded from ANOVA analyses due to the limited species number.

**Supplementary Table S4.** Model outputs for ordinary least squares (OLS) and phylogenetic generalized least squares (PGLS) regressions of the vein thickness vs. vein density (VT vs. VD) across all species, trees and shrubs, respectively.

|  | | Slope | Intercept | *F* value | *R^2^* | *P* |
| --- | --- | --- | --- | --- | --- | --- |
| All | **OLS** | **-0.62** | **1.90** | **32.31** | **0.21** | **<0.001** |
|  | PGLS | -0.23 | 1.88 | 1.187 | 0.01 | 0.279 |
| Tree | **OLS** | **-0.47** | **1.76** | **16.67** | **0.12** | **<0.001** |
|  | PGLS | 0.10 | 81.74 | 0.005 | <0.001 | 0.944 |
| Shrub | **OLS** | **-0.65** | **1.96** | **28.46** | **0.24** | **<0.001** |
|  | **PGLS** | **-4.84** | **65.15** | **12.36** | **0.19** | **<0.001** |

Statistically significant relationships are in bold (*P* < 0.05). VT and VD data were log_10_-transformed prior to analysis.

**Supplementary Table S5.** Standardized major axis (SMA) regression analyses for the relationships between vein thickness (VT) and density (VD) between trees and shrubs.

| Growth  form | *n* | *R^2^* | *P* | Slope | Slope CI | Intercept | Heterogeneity  of slope | Common  slope | Shift in  elevation |
| --- | --- | --- | --- | --- | --- | --- | --- | --- | --- |
| Tree | 40 | 0.29 | <0.01 | -1.37 | (-1.80, -1.04) | 2.55a | *P* = 0.794 | -1.33 | *P* = 0.008 |
| Shrub | 53 | 0.04 | 0.147 | -1.30 | (-1.70, -1.00) | 2.36b |  |  |  |

Note: *n*, species number. 95% confidence intervals (CI) of SMA slopes are shown in parentheses. SMA tests for common slopes revealed no significant differences between the two plant groups (*P* > 0.05). Hence, the common slope was given, and difference in elevation (i.e. y-axis intercept) between SMAs were then tested. VT and VD data were log_10_-transformed prior to analysis.

**Supplementary Table S6.** Variance component analysis of vein traits across all species, trees, and shrubs.

|  | Variance component (%) | | | | | Percentage variation at and above order level | Percentage variation at and above family level |
| --- | --- | --- | --- | --- | --- | --- | --- |
| Trait | Clade | Clade/  order | Clade/order/family  /species | Site | Residual |  |  |
| **All** |  |  |  |  |  |  |  |
| VD | 42.6 | <0.01 | 27.8 | 3.2 | 26.4 | 42.6 | 70.3 |
| VT | 54.7 | <0.01 | 6.7 | <0.01 | 38.7 | 54.7 | 61.3 |
| VV | 40.8 | 2.9 | 6.8 | <0.01 | 49.5 | 43.7 | 50.5 |
| **Tree** |  |  |  |  |  |  |  |
| VD | 65.6 | <0.01 | 20.9 | <0.01 | 13.4 | 65.6 | 86.6 |
| VT | 74.4 | <0.01 | 3.4 | <0.01 | 22.2 | 74.4 | 77.8 |
| VV | 62.4 | <0.01 | 4.1 | <0.01 | 33.4 | 62.4 | 66.6 |
| **Shrub** |  |  |  |  |  |  |  |
| VD | 16.0 | <0.01 | 20.8 | 6.3 | 56.9 | 16.0 | 36.8 |
| VT | <0.01 | 21.8 | 0.0 | <0.01 | 78.2 | 21.8 | 21.8 |
| VV | <0.01 | 12.8 | 4.3 | <0.01 | 82.9 | 12.8 | 17.1 |

All the trait data are log_10_-transformed prior to analysis and their abbreviations are given in Table S3.

**Supplementary Table S7.** Data of leaf vein traits for 93 woody species. PFT: plant functional type, including conifer and woody angiosperms (deciduous tree, deciduous shrub, and evergreen shrub). VD, vein density (mm mm^-2^); VT, vein thickness (μm); VV, vein volume per area (mm^3^ mm^-2^×10^-3^).

| No. | Species name | PFT | VD | VT | VV |
| --- | --- | --- | --- | --- | --- |
| 1 | *Abies_fargesii* | Conifer | 1.29 | 182.74 | 32.07 |
| 2 | *Acer_caesium* | Deci Tree | 5.71 | 56.16 | 14.14 |
| 3 | *Acer_cappadocicum* | Deci Tree | 6.06 | 40.44 | 7.78 |
| 4 | *Acer_davidii* | Deci Tree | 4.68 | 27.86 | 2.97 |
| 5 | *Acer_pictum* | Deci Tree | 5.89 | 26.08 | 3.14 |
| 6 | *Acer_robustum* | Deci Tree | 5.41 | 17.75 | 1.34 |
| 7 | *Acer_sterculiaceum* | Deci Tree | 3.89 | 45.00 | 6.19 |
| 8 | *Acer_tataricum* | Deci Tree | 4.81 | 50.21 | 9.52 |
| 9 | *Akebia_trifoliata* | Deci shrub | 5.51 | 27.29 | 3.23 |
| 10 | *Amelanchier_sinica* | Deci Tree | 8.08 | 36.79 | 8.59 |
| 11 | *Berberis_amurensis* | Deci shrub | 4.83 | 42.28 | 7.11 |
| 12 | *Betula_albosinensis* | Deci Tree | 7.85 | 38.90 | 9.33 |
| 13 | *Betula_utilis* | Deci Tree | 7.87 | 48.13 | 14.02 |
| 14 | *Buckleya_lanceolate* | Deci shrub | 4.30 | 20.41 | 1.41 |
| 15 | *Buddleja_albiflora* | Deci shrub | 9.04 | 24.82 | 4.37 |
| 16 | *Campylotropis_macrocarpa* | Deci shrub | 7.24 | 29.23 | 4.86 |
| 17 | *Carpinus_cordata* | Deci Tree | 7.00 | 43.11 | 10.22 |
| 18 | *Castanea_mollissima* | Deci Tree | 6.56 | 9.36 | 0.45 |
| 19 | *Celastrus_orbiculatus* | Deci shrub | 7.56 | 44.44 | 11.72 |
| 20 | *Celtis_sinensis* | Deci Tree | 8.45 | 26.07 | 4.51 |
| 21 | *Clerodendrum_trichotomum* | Deci shrub | 5.13 | 13.74 | 0.76 |
| 22 | *Cornus_alba* | Deci shrub | 5.85 | 27.35 | 3.44 |
| 23 | *Cornus_controversa* | Deci Tree | 6.41 | 15.31 | 1.18 |
| 24 | *Cornus_hemsleyi* | Deci shrub | 8.37 | 14.95 | 1.47 |
| 25 | *Cornus_kousa* | Deci Tree | 5.34 | 18.17 | 1.38 |
| 26 | *Crataegus_hupehensis* | Deci Tree | 6.16 | 43.79 | 9.28 |
| 27 | *Crataegus_pinnatifida* | Deci Tree | 4.29 | 15.78 | 0.84 |
| 28 | *Daphne_giraldii* | Deci shrub | 6.52 | 36.18 | 6.70 |
| 29 | *Decaisnea_insignis* | Deci shrub | 4.44 | 19.69 | 1.35 |
| 30 | *Deutzia_taibaiensis* | Deci shrub | 4.33 | 24.77 | 2.09 |
| 31 | *Diospyros_lotus* | Deci Tree | 8.21 | 12.50 | 1.01 |
| 32 | *Elaeagnus_umbellata* | Deci shrub | 3.66 | 18.44 | 0.98 |
| 33 | *Eleutherococcus_giraldii* | Deci shrub | 5.69 | 16.99 | 1.30 |
| 34 | *Eleutherococcus_setchuenensis* | Deci shrub | 3.45 | 19.44 | 1.03 |
| 35 | *Euonymus_alatus* | Deci shrub | 6.23 | 41.10 | 8.26 |
| 36 | *Euonymus_phellomanus* | Deci shrub | 3.90 | 23.72 | 1.72 |
| 37 | *Fraxinus_insularis* | Deci Tree | 3.77 | 40.85 | 4.94 |
| 38 | *Fraxinus_stylosa* | Deci Tree | 5.91 | 18.48 | 1.58 |
| 39 | *Juglans_mandshurica* | Deci Tree | 14.59 | 16.08 | 2.96 |
| 40 | *Juglans_regia* | Deci Tree | 8.66 | 22.19 | 1.93 |
| 41 | *Juniperus_pingii* | Conifer | 4.10 | 60.08 | 11.61 |
| 42 | *Kalopanax_septemlobus* | Deci Tree | 3.73 | 39.95 | 4.67 |
| 43 | *Kerria_japonica* | Deci shrub | 5.55 | 56.50 | 13.93 |
| 44 | *Koelreuteria_paniculata* | Deci Tree | 8.73 | 14.25 | 1.39 |
| 45 | *Larix_potaninii* | Conifer | 3.90 | 96.24 | 36.49 |
| 46 | *Lindera_obtusiloba* | Deci Tree | 6.51 | 35.33 | 6.38 |
| 47 | *Litsea_pungens* | Deci Tree | 4.49 | 24.38 | 2.10 |
| 48 | *Lonicera_fragrantissima* | Deci shrub | 4.72 | 61.12 | 13.84 |
| 49 | *Lonicera_hispida* | Deci shrub | 6.21 | 25.93 | 3.67 |
| 50 | *Lonicera_stephanocarpa* | Deci shrub | 5.79 | 41.03 | 8.00 |
| 51 | *Lonicera_tangutica* | Deci shrub | 9.15 | 25.20 | 4.56 |
| 52 | *Lonicera_tragophylla* | Deci shrub | 6.64 | 18.08 | 1.70 |
| 53 | *Morus_australis* | Deci shrub | 8.89 | 10.28 | 0.74 |
| 54 | *Padus_racemosa* | Deci Tree | 5.69 | 15.43 | 1.06 |
| 55 | *Parthenocissus_laetevirens* | Deci shrub | 4.85 | 15.81 | 0.95 |
| 56 | *Philadelphus_incanus* | Deci shrub | 3.77 | 26.22 | 2.08 |
| 57 | *Populus_tremula* | Deci Tree | 10.50 | 29.23 | 7.05 |
| 58 | *Potentilla_glabra* | Deci shrub | 9.24 | 18.82 | 2.57 |
| 59 | *Prunus_tomentosa* | Deci shrub | 6.28 | 25.13 | 2.61 |
| 60 | *Pyrola_calliantha* | Deci shrub | 3.14 | 12.44 | 0.38 |
| 61 | *Quercus_aliena* | Deci Tree | 8.21 | 34.05 | 7.47 |
| 62 | *Rhamnus_davurica* | Deci shrub | 5.00 | 24.03 | 2.27 |
| 63 | *Rhododendron_capitatum* | Ever shrub | 5.87 | 38.45 | 7.28 |
| 64 | *Rhododendron_clementinae* | Ever shrub | 7.19 | 24.61 | 3.50 |
| 65 | *Rhododendron_concinnum* | Ever shrub | 4.81 | 35.18 | 4.68 |
| 66 | *Rhus_potaninii* | Deci Tree | 6.77 | 32.21 | 5.52 |
| 67 | *Ribes_giraldii* | Deci shrub | 4.60 | 14.60 | 0.77 |
| 68 | *Ribes_glaciale* | Deci shrub | 4.39 | 24.64 | 2.05 |
| 69 | *Rosa_omeiensis* | Deci shrub | 8.77 | 12.07 | 1.06 |
| 70 | *Rosa_tsinglingensis* | Deci shrub | 5.05 | 20.78 | 1.71 |
| 71 | *Rubus_flosculosus* | Deci shrub | 6.83 | 12.79 | 0.93 |
| 72 | *Rubus_glabricarpus* | Deci shrub | 4.02 | 17.96 | 1.02 |
| 73 | *Salix_babylonica* | Deci Tree | 9.43 | 33.47 | 8.65 |
| 74 | *Salix_cupularis* | Deci shrub | 6.65 | 41.70 | 9.86 |
| 75 | *Salix_taipaiensis* | Deci shrub | 10.17 | 19.35 | 2.99 |
| 76 | *Sambucus_williamsii* | Deci shrub | 4.56 | 23.77 | 2.02 |
| 77 | *Schisandra_sphenanthera* | Deci shrub | 5.99 | 30.86 | 4.48 |
| 78 | *Smilax_stans* | Deci shrub | 3.58 | 45.45 | 5.80 |
| 79 | *Sorbaria_sorbifolia* | Deci shrub | 7.55 | 21.55 | 2.75 |
| 80 | *Sorbus_alnifolia* | Deci Tree | 6.23 | 23.21 | 2.64 |
| 81 | *Sorbus_discolor* | Deci Tree | 7.59 | 16.76 | 1.68 |
| 82 | *Sorbus_folgneri* | Deci Tree | 3.72 | 32.25 | 3.04 |
| 83 | *Sorbus_sp.* | Deci Tree | 8.19 | 39.78 | 10.17 |
| 84 | *Sorbus_tianschanica* | Deci shrub | 5.70 | 37.88 | 6.43 |
| 85 | *Spiraea_alpina* | Deci shrub | 10.48 | 13.54 | 1.51 |
| 86 | *Spiraea_japonica* | Deci shrub | 6.50 | 13.20 | 0.89 |
| 87 | *Spiraea_salicifolia* | Deci shrub | 6.91 | 40.37 | 8.84 |
| 88 | *Symplocos_paniculata* | Deci shrub | 9.70 | 19.13 | 2.79 |
| 89 | *Tilia_chinensis* | Deci Tree | 11.14 | 23.04 | 4.65 |
| 90 | *Toxicodendron_vernicifluum* | Deci Tree | 9.07 | 15.16 | 1.64 |
| 91 | *Viburnum_dilatatum* | Deci shrub | 4.78 | 29.75 | 3.32 |
| 92 | *Vitis_piasezkii* | Deci shrub | 5.32 | 20.51 | 1.76 |
| 93 | *Zanthoxylum_bungeanum* | Deci Tree | 10.01 | 20.44 | 3.51 |

# Figure


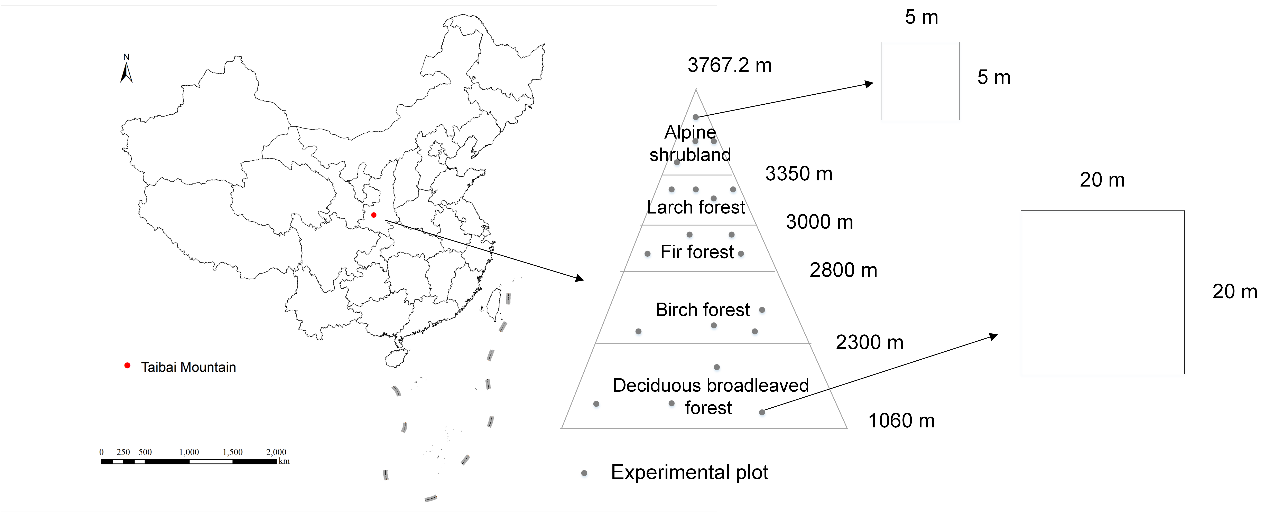


**Supplementary Figure S1.** Study area and sampling sites on the northern slope in Taibai Mountain. Five sampling sites with four experimental plots (20 × 20 m plot for forest sites, 5 × 5 m plot for shrubland) at each vertical vegetation belt were established along the altitudinal gradients.
